# Injury-Related Unsafe Behavior Among Households from Different Socioeconomic Strata in Pune City

**DOI:** 10.4103/0970-0218.58387

**Published:** 2009-10

**Authors:** Roksana Mirkazemi, Anita Kar

**Affiliations:** Interdisciplinary School of Health Sciences, University of Pune, Pune 411 007, India

**Keywords:** India, pattern of behavior, unintentional injury, urban households

## Abstract

**Introduction:**

Behavior pattern influences the risk of unintentional injuries. This study was conducted to identify the pattern of household unsafe behavior in different socioeconomic strata, in Pune city, India.

**Materials and Method:**

Population-based, cross-sectional study. Behaviors influencing the risk of burn, poisoning, drowning, and road traffic injuries were questioned from 200 randomly selected households.

**Results:**

Nearly 28% of the households did not have a separate kitchen, 37.5% cooked at the ground level, 33.5% used a kerosene pressure stove, 12% used unprotected open fire as a source of warmth in winter, and 34.5% stored inflammable substances at home. Ninety one percent of the households reported storing poisonous chemicals in places that could not be locked. In 68.3% of the households with children below five years, these chemicals were kept in places accessible to children. Nearly 21% of the individuals, who could swim, did so in unsafe places and 25.2% of them were not trained in swimming. In 35.5% of the households, children used streets as playgrounds. Among all two-wheeled vehicle riders, 35.6% reported not having a helmet and 57.7% of those who had a helmet did not use it regularly. Socioeconomic status was strongly associated with the unsafe behaviors related to burns, drowning, and road traffic injuries.

**Conclusion:**

The study identifies the sociocultural and behavioral factors leading to unsafe behaviors, placing individuals at risk of unintentional injuries, which can be used as a first step toward prevention.

## Introduction

Unintentional injuries are a major public health problem worldwide, but receive minimum attention in developing countries. In these countries, urban development in transition exposes individuals and households to unsafe environments. In India, the unintentional injury occurrence rate was reported to be 110 cases per 1000 individuals per year, which was more than 15 times that of intentional injury occurrence rates (seven cases per 1000 individuals per year).(1) Road traffic injuries, burns, poisonings, and drowning were responsible for 40% of the total injury occurrence in India.(1) The annual burn-related death was reported at 35,000 cases.(2) The majority of burns (77.5%) occurred at home in the kitchen and among females.(3) The risk factors associated with burns included cooking on open fires, the practice of low-level cooking, leakage, and explosion of pressure stoves, use of unprotected open fires to keep warm during winter, storage of inflammable substances, and housing that is located in slums and congested areas.(4–6) In India, case fatality rate of poisoning varied from 2 to 5%. Maximum cases of poisoning were due to household agents like kerosene, drugs, and pesticides via the oral route. Storage in nonstandard containers and the storage of poisons at the ground level were other factors increasing the risk of poisoning.(2,4,6,7) India has the highest female drowning mortality rate in the world with more than 22,000 fatalities in 1999.(8) The major cause of drowning was due to submergence in rivers, lakes, or drainage systems.(4,6) Deaths due to road traffic injuries exceeded 80,000 persons per year in India.(9) Vulnerable road users were pedestrians, riders and pillion riders of motorized two-wheeled vehicles, and bicyclists.(6,9–12)

Knowledge about behavioral patterns of households is required for any preventive plan, to reduce the risk of injury and enhance behavioral changes. Among many factors influencing this pattern of behavior, socioeconomic status is an identified risk factor for unintentional injuries.(13–15) This population-based study was conducted to identify the pattern of household behaviors, which may influence the risk of burn, poisoning, drowning, and road traffic injuries in different socioeconomic strata of the society in the city of Pune, in India.

## Materials and Methods

Data collection for a population-based study to determine the burden, pattern, and risk factors for unintentional injuries was conducted in Pune, between March 2007 and April 2008. In this study, a sample of 2100 households was randomly selected through multistage, stratified, cluster random sampling. Ten percent of this sample population (200 households) was randomly selected from within 10 administrative wards of Pune city (20 households per ward). The pattern of household behavior that influences the risk of unintentional injuries was elicited from a household member, usually the head of the household or spouse of the head of the household. A semi-structured questionnaire was used to collect information about the sociodemographic status of the households and the pattern of household behaviors, which may increase the risk of the four most prevalent unintentional injuries, that is, burn, poisoning, drowning, and road traffic injuries. The socioeconomic classification was based on the revised Kuppuswamy score.(16) Classification of the type of housing was based on the definition of Census 2001.(17) Chi Square test was performed using SPSS version 13 to measure the association between variables and socioeconomic status of households/individuals. Cases of non-response or not applicable were not included in the Chi Square test. Risk factors were selected based on previous published literature.(2–7,9–11)

## Results

### Description of socioeconomic status of households

Out of 200 households interviewed, maximum households belonged to the lower socioeconomic strata (38.5%, 77 households). Twenty-five (12.5%) households resided in slums and two households (1%) were homeless. Eighteen (9%) households resided in semi-permanent houses and two (1.0%) in temporary houses. Thirty-three households (16.5%) had only one room as dwelling area, 62 households (31.0%) lived in homes with a density of two to five individuals per room, and five households (2.5%) lived in homes with a density between six to 10 individuals per room [Table 1].

**Table 1:** Socioeconomic status of the households, household behaviors influencing the risk of injuries, household behaviors influencing the risk of burn

Variable	Number (%)
Socioeconomic score (n = 200)	
Lower	77 (38.5)
Middle	53 (26.5)
Upper	70 (35.0)
Density of individual/room (n = 200)	
≤ 2	130 (65.0)
2 to 5	62 (31.0)
6 to 10	5 (2.5)
> 10	1 (0.5)
Not applicable	2 (1.0)
Residence (n = 200)	
Slum	25 (12.5)
Non Slum	137 (86.5)
Not applicable	2 (1.0)
Type of housing (n = 200)	
Permanent	180 (90.0)
Temporary	2 (1.0)
Semi permanent	18 (9.0)

Unsafe behaviors increasing the risk of burn injury was cooking in the living area (27.8%, 55 households), use of kerosene pressure stove in addition to gas stove (27.5%, 55 households), cooking at ground level (27.5%, 55 households), using open fire as source of warmth during winter (12.0%, 24 households), and storage of inflammable substances at home (34.5%, 69 households). Only one household reported having a fire extinguisher in working condition and four households reported availability of fire extinguisher in the apartment block. Low socioeconomic status was strongly associated with behaviors and situations that increased the risk of burns, like cooking in the living area (*P* = 0.000), using unsafe cooking equipment (pressure stove or unprotected open fire) (*P* = 0.000), cooking at the ground level (*P* = 0.000), storing inflammable substances at home (*P* = 0.000), and using unprotected open fire for warmth during cold season (*P* = 0.000) [Table 2].

**Table 2:** Household behavior influencing the risk of unintentional injuries

	Lower	Middle	Upper	*P* value	Total
Burn					
Cooking in the living area (n = 200)	47 (62.7)	7 (13.2)	1 (1.4)	0.000	55 (27.8)
Cooking equipment (n = 200)				0.000	
Gas stove	26 (33.8)	42 (79.2)	65 (92.9)		133 (66.5)
Kerosene pressure stove	7 (9.1)	0 (0.0)	0 (0.0)		7 (3.5)
Gas / Kerosene stove	39 (50.6)	11 (20.8)	5 (7.1)		55 (27.5)
Open fire	5 (6.5)	0 (0.0)	0 (0.0)		5 (2.5)
Cooking at the ground level (n = 200)	57 (74.0)	15 (28.3)	3 (4.3)	0.000	75 (37.5)
Storing Inflammable substances (n = 200)	48 (62.3)	14 (26.4)	7 (10.0)	0.000	69 (34.5)
Using open fire for warming (n = 200)	20 (26.0)	3 (5.7)	1 (1.4)	0.000	24 (12.0)
Poisoning					
Presence of poisoning agent at home (n = 200)	66 (85.7)	50 (94.3)	66 (94.3)	0.118	182 (91.0)
Poisoning agent accessible to < 5 children (n = 60)	20 (74.1)	10 (62.5)	11 (64.7)	0.682	41 (68.3)
Road traffic injuries					
Street as playing ground (n = 93)	23 (54.8)	7 (30.4)	3 (10.7)	0.001	33 (35.5)
Mode of transport (n = 921)				0.000	
Four wheeler	0 (0.0)	1 (0.4)	72 (25.2)		73 (7.9)
Two wheeler / two wheeler + other	85 (21.9)	115 (46.7)	143 (50.0)		343 (37.2)
Public transport	211 (54.2)	102 (41.5)	66 (23.1)		379 (41.2)
Walk / cycle	93 (23.9)	28 (11.4)	5 (1.7)		126 (13.7)
Driving / riding a vehicle (n = 921)	49 (12.6)	87 (35.4)	135 (47.2)	0.000	271 (29.4)
Driving without license (n = 240)*	1 (2.6)	1 (1.4)	2 (1.5)	0.879	4 (1.7)
Having no helmet (n = 191)	18 (54.5)	29 (42.0)	21 (23.6)	0.002	68 (35.6)
No regular use of helmet (n = 123)	9 (60.0)	29 (72.5)	33 (48.5)	0.051	71 (57.7)
Number of pillion rider >1 (n = 191)	20 (60.6)	24 (34.8)	29 (32.6)	0.014	73 (38.2)
Drowning					
Presence of unprotected water surface (n = 200)	26 (33.8)	11 (20.8)	28 (40.0)	0.075	65 (32.5)
Having swimming habit (n = 936)	19 (16.5)	43 (37.4)	53 (46.1)	0.000	115 (12.3)
Swimming in unsafe places (n = 115)	16 (84.2)	8 (18.6)	0 (0.0)	0.000	24 (20.9)
Being an untrained swimmer (n = 115)	8 (42.1)	19 (44.2)	2 (3.8)	0.000	29 (25.2)

* In 31 cases, license was not applicable as the vehicle was a bicycle

### Household behaviors influencing the risk of poisoning at home

Presence of chemicals, which could be potential poisons (kerosene, phenyl, drugs and pesticides), in unlocked storage places was reported by 91.0% of the households (n = 182). Seventy-one households (35.5%) reported storing kerosene at home in nonstandard containers usually meant for beverage or food items. Among 60 households with children below five years, 41 households (68.3%) reported that these chemicals were accessible to children. Storage of poisonous chemicals at home was not associated with the socioeconomic status of the households. However, households from the higher socioeconomic strata stored safer forms of chemicals, for example, insecticide spray (50%), while households from lower socioeconomic strata used unsafe forms like insecticides in tablet or liquid form (86.8%). There was no significant association between socioeconomic status and storage of poisonous chemicals at places accessible to children below five years (*P* = 0.682) [Table 2].

### Household behaviors influencing the risk of road traffic injuries

Unsafe behaviors increasing the risk of road traffic injury were children playing in the streets (35.5%, 33 households), using motorized two-wheeled vehicles (37.2%, 343 individuals), being pedestrians (41.2%, 379 individuals), driving without having a license (1.7%, 4 drivers), not having a helmet among riders of two-wheeled vehicles (35.6%, 68 riders), irregular use of helmet despite possessing one (57.7%, 71 riders), and more than the permitted number of pillion riders (38.2%, 73 riders). Low socioeconomic status was significantly associated with the unsafe outdoor playing area of children (*P* = 0.001), since use of streets for playing was reported in children of 54.8% of the households belonging to lower socioeconomic strata, but only from 10.7% of households that belonged to the upper socioeconomic strata. Not having a helmet (*P* = 0.002) and carrying more than one pillion rider (*P* = 0.014) was strongly associated with low socioeconomic status. Among individuals belonging to the lower socioeconomic strata, 54.5% (n = 18) did not have a helmet, while this proportion was 23.6% (n = 21) among individuals belonging to the upper socioeconomic strata. In the lower socioeconomic strata, 60.6% (n = 20) reported carrying more than one pillion rider, while this proportion was 32.6% (n = 29) in the upper socioeconomic strata. There was no significant association between socioeconomic status of individuals and driving without license (*P* = 0.879) or irregular use of helmet (*P* = 0.051) [Table 2].

### Factors influencing the risk of drowning

Presence of unprotected water surface in the vicinity (< 1 km) of the living area was reported by 32.5% of households (n = 65). Unsafe behaviors increasing the risk of drowning were swimming in places without lifeguard or safety devices (20.9%, n = 24) and swimming without being trained in swimming (25.2%, n = 29). There was no association between the socioeconomic status and presence of unprotected water surface in the vicinity of the living area (*P* = 0.075). There was a strong association between the socioeconomic status and swimming in unsafe places where lifeguard and safety devices were not available (*P* = 0.000). Among individuals belonging to the upper socioeconomic strata, who reported the habit of swimming, only 3.8% had not received formal training for swimming, but this proportion was 42.1% among individuals belonging to the lower socioeconomic strata [Table 2].

## Discussion

A bibliographic search on unintentional injuries in India yields very few reports on injury-related, unsafe behavior. The available studies relate to specific injuries, for example, risky behavior of drivers of motorized two-wheeled vehicles,(12) hand injury in sugarcane crushers,(18) or alcohol use and road traffic injuries.(19) This study is the first population-based study, which shows the extent of injury-related unsafe behavior among households in India. In developed nations, studies on injury-related behavioral risk, provides the baseline data for the design and evaluation of preventive measures. In the United States, for example, injury-related behavioral risk factor surveillance, consisting of monthly telephone interviews, are in existence, in order to monitor the behavior-related risk factors for unintentional injuries.(20)

In this study, the underlying causes of unsafe behavior could be ascribed to socioeconomic and cultural factors, lack of awareness, lack of or poor urban infrastructure, and lack of proactive preventive measures by the government and public health agencies. Unsafe cooking practices could be related not only to the traditional Indian practice of floor level cooking, but also to poverty, which forces families to use unprotected open fire or a kerosene pressure stove for cooking. Irregular supply of cooking gas was also associated with at least one-third of the households using pressure kerosene stoves as a back-up cooking device. Thus, measures to ensure proper distribution of cooking gas along with price subsidies for the poor, could be an active preventive measure to reduce the risk of burns. Education about the importance of tabletop cooking is another preventive measure for increasing safe cooking practices. Rare reports of fire extinguishers at homes of even households belonging to the high strata of society, suggests the need for legislation to make fire extinguishers mandatory, at least in apartment blocks.

Lack of awareness about the risk of poisoning was evident from the widespread unsafe practice of storing poisonous chemicals at places accessible to children, even among households from the higher socioeconomic strata. In addition to education, public health agencies can play an active role by promoting awareness on proper storage of poisonous substances and making use of childproof containers mandatory.

Poor urban infrastructure like presence of unprotected water bodies and lack of safe playgrounds increase the risk of drowning and road traffic injuries, especially for children. High rate of pedestrian and public transport use, especially among the poor, highlights the need for improvement of urban infrastructure, especially in the face of the rapid population increase, including in-migration into cities. Not having a helmet was reported mostly among the poor. Creating awareness to increase the risk perception along with compulsory distribution of helmets at the time of selling motorized two-wheeled vehicles could be an intervention to support the safety of the poor. Low compliance to helmet use, in a situation where motorized two-wheeled vehicles were used by more than 35% of the households, shows the importance of legislation, education, and enforcement. One of the main reasons cited for not using a helmet was discomfort. Thus changing the design of helmets to give better side view could be effective in increasing the rate of helmet usage.

## Conclusion

Injury-related unsafe behavior is widely prevalent among households, with greater prevalence in the lower socioeconomic strata. Interventions aimed at behavior change have to be considered concomitantly with the necessity of improving and providing safer infrastructure in urban environments.
